# Draft Genome Sequence of *Sphingobium* sp. Strain C100, a Polycyclic Aromatic Hydrocarbon-Degrading Bacterium from the Deep-Sea Sediment of the Arctic Ocean

**DOI:** 10.1128/genomeA.01210-13

**Published:** 2014-01-30

**Authors:** Chunming Dong, Xiuhua Bai, Qiliang Lai, Yanrong Xie, Xin Chen, Zongze Shao

**Affiliations:** a Key Laboratory of Marine Genetic Resources, Third Institute of Oceanography, State Oceanic Administration, Xiamen, China; b State Key Laboratory Breeding Base of Marine Genetic Resources, Xiamen, China; c Key Laboratory of Marine Genetic Resources of Fujian Province, Xiamen, China; d Life Science College, Xiamen University, Xiamen, Fujian, China

## Abstract

*Sphingobium* sp. strain C100 was isolated from a polycyclic aromatic hydrocarbon (PAH)-degrading consortium from the deep-sea sediment of the Arctic Ocean. It can degrade two- to four-ring PAHs at 25°C. Here we present the draft genome sequence of this strain, which is 4,776,810 bp with a G+C content of 63.9%.

## GENOME ANNOUNCEMENT

Members of the *Sphingobium* genus are Gram-negative, nonsporulating, rod-shaped aerobic bacteria with yellow or whitish-brown colonies (1). They have been isolated from a wide variety of environments and show the ability to degrade many kinds of xenobiotics, such as aromatic and chloroaromatic compounds (2–4). Here, we present the draft genome sequence of a *Sphingobium* bacterium, strain C100. The bacterium was isolated from a polycyclic aromatic hydrocarbon (PAH)-degrading consortium, which was enriched from the deep-sea sediment of the Makarov Basin (170°29′W, 87°04′N; water depth of 4,000 m) in the Arctic Ocean, using a PAH mixture of naphthalene, phenanthrene, and pyrene as the sole carbon and energy source. Our previous studies indicated that this strain could degrade naphthalene, 2-methyl naphthalene, fluorene, acenaphthene, phenanthrene, anthracene, and fluoranthene at 25°C (unpublished data).

Genomic DNA was purified from strain C100 with an AxyPrep bacterial genomic DNA miniprep kit (Axygen) according to the manual instructions. The genome was sequenced using the Genome Analyzer IIx system at Shanghai Majorbio Bio-pharm Technology Co., Ltd. (Shanghai, China), which produced 955 Mbp paired-end reads of 80 bp with an insert size of 300 bp. Approximately 753-Mbp high-quality reads were assembled with SOAPdenovo v 1.05 (5). The final genome assembly has 157-fold coverage and contains 219 scaffolds composed of 170 contigs (longer than 1,000 bp) with a total size of 4,776,810 bp, an *N*_50_ contig length of 46,449 bp, and a mean G+C content of 63.9%.

Gene prediction and annotation of the draft genome were carried out using the NCBI Prokaryotic Genome Annotation Pipeline (PGAP) (http://www.ncbi.nlm.nih.gov/genomes/static/Pipeline.html) (6). In total, 4,634 genes were predicted, 4,513 of which are protein-coding genes, and 61 RNAs; 60 pseudogenes were also identified. The majority of the protein-coding genes (3,268, 70.5%) were assigned a putative function, while the remaining ones were annotated as hypothetical proteins. In addition, a clustered regularly interspaced short palindromic repeat (CRISPR) array was found in the draft genome.

We particularly analyzed genes possibly responsible for the degradation of PAHs. At least 2 naphthalene 1,2-dioxygenase (7, 8), 6 aromatic-ring-hydroxylating dioxygenase (9), and 12 ferredoxin-related coding genes, which are responsible for the first catalytic reaction of PAH degradation (10), were found in the draft genome. During the degradation of PAHs, catechol, protocatechuate, gentisate, benzoate, and phthalate are the important intermediate metabolites (10). Congruently, one catechol 2,3-dioxygenase, two protocatechuate 3,4-dioxygenase, three protocatechuate 4,5-dioxygenase, one gentisate 1,2-dioxygenase, and two benzoate 1,2-dioxygenase genes and one phthalate 4,5-dioxygenase gene were also found in the genome. In addition, six cytochrome P450 genes were present in the genome. A previous study demonstrated that cytochrome P450 was involved in the degradation of PAHs (11). The genome sequence of *Sphingobium* sp. strain C100 will be helpful in understanding the PAH-degrading mechanism of the *Sphingobium* species.

### Nucleotide sequence accession number.

The annotated draft genome sequence was deposited in DDBJ/EMBL/GenBank under accession no. AYOY00000000. The version described in this paper is the first version.
